# Bayesian dynamical system analysis of the effects of methylphenidate in children with attention-deficit/hyperactivity disorder: a randomized trial

**DOI:** 10.1038/s41386-023-01668-3

**Published:** 2023-07-25

**Authors:** Weidong Cai, Yoshifumi Mizuno, Akemi Tomoda, Vinod Menon

**Affiliations:** 1 Department of Psychiatry & Behavioral Sciences, Stanford University School of Medicine, Stanford, USA; 2 Wu Tsai Neuroscience Institute, Stanford University, Stanford, USA; 3 Research Center for Child Mental Development, University of Fukui, Fukui, 910-1193 Japan; 4 Division of Developmental Higher Brain Functions, United Graduate School of Child Development, University of Fukui, Fukui, 910-1193 Japan; 5 Department of Child and Adolescent Psychological Medicine, University of Fukui Hospital, Fukui, 910-1193 Japan; 6 Department of Neurology & Neurological Sciences, Stanford University School of Medicine, Stanford, USA

**Keywords:** ADHD, Attention

## Abstract

Methylphenidate is a widely used and effective treatment for attention-deficit/hyperactivity disorder (ADHD), yet the underlying neural mechanisms and their relationship to changes in behavior are not fully understood. Specifically, it remains unclear how methylphenidate affects brain and behavioral dynamics, and the interplay between these dynamics, in individuals with ADHD. To address this gap, we used a novel Bayesian dynamical system model to investigate the effects of methylphenidate on latent brain states in 27 children with ADHD and 49 typically developing children using a double-blind, placebo-controlled crossover design. Methylphenidate remediated greater behavioral variability on a continuous performance task in children with ADHD. Children with ADHD exhibited aberrant latent brain state dynamics compared to typically developing children, with a single latent state showing particularly abnormal dynamics, which was remediated by methylphenidate. Additionally, children with ADHD showed brain state-dependent hyper-connectivity in the default mode network, which was also remediated by methylphenidate. Finally, we found that methylphenidate-induced changes in latent brain state dynamics, as well as brain state-related functional connectivity between salience and default mode networks, were correlated with improvements in behavioral variability. Taken together, our findings reveal a novel latent brain state dynamical process and circuit mechanism underlying the therapeutic effects of methylphenidate in childhood ADHD. We suggest that Bayesian dynamical system models may be particularly useful for capturing complex nonlinear changes in neural activity and behavioral variability associated with ADHD. Our approach may be of value to clinicians and researchers investigating the neural mechanisms underlying pharmacological treatment of psychiatric disorders.

## Introduction

Attention-deficit/hyperactivity disorder (ADHD) is a highly prevalent neurodevelopmental disorder that affects approximately 5–10% of children and adolescents worldwide [1]. ADHD is characterized by deficits in attention and cognitive control, which can lead to difficulties in focusing on relevant information and resisting distractions [2–4]. These impairments can have a negative impact on learning and behavior in the classroom, and may contribute to academic and social problems later in life [5, 6]. Methylphenidate is a widely used first-line medication for alleviating symptoms of inattention in children with ADHD [7] and it improves their academic performance [8]. Despite its widespread and effective use in clinical practice, the precise brain circuit mechanisms by which methylphenidate improves attention and behavioral variability in children with ADHD are poorly understood [9]. Given the importance of understanding brain dynamics in supporting cognitive functions [10–13], there is a growing interest in utilizing dynamical system models [14] to investigate the effects of methylphenidate in children with ADHD.

One of the most robust behavioral phenotypes associated with attentional deficits in childhood ADHD is intra-individual response variability (IIRV), which measures dynamic fluctuations in trial-to-trial performance [15, 16]. Increased IIRV has been linked to poor attention and cognitive control [17, 18]. Although the etiology of such behavioral instability is not yet clear, researchers have suggested a link with abnormal fluctuations in brain states, leading to sluggish information processing and poor decision making [19, 20]. Findings from a recent study are in favor of this hypothesis, showing that engaging in non-optimal brain state is associated with increased IIRV in children [11]. However, it remains unknown whether methylphenidate improves attention in children with ADHD by normalizing aberrant brain state dynamics and IIRV.

Bayesian dynamical system models are now increasingly used to investigate latent brain states and dynamic brain circuits associated with them [11, 13, 21, 22]. This approach allows for the investigation of the complex and nonlinear dynamics of brain activity over time, and can uncover dysfunctional brain circuits that are difficult to capture using conventional linear models [13]. Moreover, these models may be better placed to capture aberrancies in behavioral fluctuations of attention which are a key phenotypic feature of ADHD [11]. Crucially, Bayesian dynamical system models could be especially valuable for studying the effects of methylphenidate on IIRV in children with ADHD, as they provide better models for linking brain and behavioral dynamics. By modeling the underlying dynamical system, this approach can provide a more precise understanding of the underlying neural mechanisms of the disorder and the effects of medication.

A substantial body of human functional neuroimaging studies has uncovered a core set of distributed regions in the salience network (SN), anchored in the anterior insula and dorsal anterior cingulate cortex, and frontal-parietal network (FPN), anchored in the dorsolateral prefrontal and posterior parietal cortex, that support attention and cognitive control [23–27], as well as the default mode network (DMN), anchored in the posterior cingulate cortex and ventromedial prefrontal cortex, that modulates attention and cognitive control [28–31]. Critically, aberrancies in these key cognitive control networks are prominent in children with ADHD [32, 33]. Abnormal activation in the SN, FPN, and DMN [34–37], aberrant intrinsic connectivity and weak task-modulated connectivity between SN, FPN and DMN regions [19, 38–42] have been reported in ADHD.

Previous studies have reported that methylphenidate alters regional activation in these brain networks during cognitive performance in individuals with ADHD [43]. A recent study has further revealed that methylphenidate reduces the difference in dynamic functional connectivity within the SN, FPN and DMN between typically developing (TD) children and children with ADHD, suggesting the medication may remediate dysfunction in the large-scale brain networks involved in attention and cognitive control [14]. However, the effects of medication on brain state dynamics and links with behavioral variability are not known. Rigorous quantitative models of brain state dynamics are needed to better understand the relationship between changes in brain state dynamics induced by medication and behavioral dynamics in children with ADHD.

In a previous study, we investigated the impact of methylphenidate on dynamic interactions between the SN, FPN and DMN [14]. We extracted network time series using independent component analysis and then employed a sliding window to determine time varying changes in connectivity. The results showed that children with ADHD exhibit aberrant dynamic connectivity between these networks, which were remediated by methylphenidate. However, the study had limitations related to the determination of critical parameters such as window length and the number of brain states, which relied on ad hoc procedures [44]. Additionally, the determination of the optimal number of components in temporal independent component analysis presented challenges, leading to non-unique options for generating network-specific time series, which were utilized as inputs in our analysis. Furthermore, previous research has not established a clear association between methylphenidate-induced changes in dynamic network interactions and alterations in intra-individual response variability (IIRV), which is a fundamental characteristic of attentional fluctuations in individuals with ADHD.

**Fig. 1:**
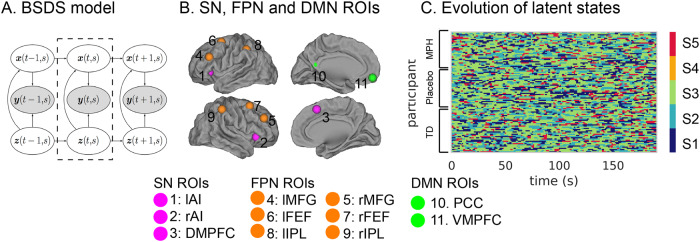
Key steps in Bayesian dynamical systems modeling. **A** Schematic illustration of the Bayesian switching linear dynamical systems (BSDS) model. **B** Regions of interest (ROIS) include key nodes in the salience (SN), frontal-parietal (FPN) and default mode networks (DMN). **C** Temporal evolution of latent brain states during rest-state fMRI of all participants. Each row represents one subject, each column represents one data point (fMRI volume). lAI left anterior insula, rAI right anterior insula; DMPFC Dorsomedial prefrontal cortex, lMFG left middle frontal gyrus, rMFG right middle frontal gyrus, lFEF left frontal eye field, rFEF right frontal eye field lIPL left inferior parietal lobule, rIPL right inferior parietal lobule, PCC Posterior cingulate cortex, VMPFC Ventromedial prefrontal cortex.

To address methodological limitations of prior studies, we used a novel Bayesian switching linear dynamic systems (BSDS) model [13] to investigate latent brain states that fluctuate over time. BSDS implements an unsupervised Bayesian learning algorithm to determine hidden (latent) brain states and dynamic state transitions automatically from observed data (Fig. 1A). Briefly, each brain state is associated with a unique dynamical process that captures time-varying activation and functional connectivity in an optimal latent subspace. Furthermore, BSDS applies a hidden Markov model to latent space variables of the observed data, resulting in a parsimonious model of generators underlying the observed data. Importantly, it does not require arbitrary moving windows or impose temporal boundaries associated with predefined task conditions, which are major limitations of existing methods for probing dynamic processes in the human brain [44]. This more rigorous computational approach has the potential to provide a deeper understanding of the relationship between changes in brain state dynamics induced by methylphenidate and cognitive functions in children with ADHD.

We investigated the impact of methylphenidate on dynamics of latent brain states and its relation with IIRV changes in children with ADHD. We conducted a randomized placebo-controlled double-blind crossover study (Figure S1) with 27 children diagnosed with ADHD and 49 TD children. Two sessions of resting-state fMRI data were acquired in each child with ADHD with a counterbalanced order in which participants were administered with methylphenidate in one session and placebo in the other. An out-of-scanner continuous performance Go/NoGo task was completed in each session. Participants were instructed to make button-press responses to a target (i.e. a triangle) and no response to a non-target stimulus (i.e. a circle or a square). Standard deviation of reaction time on target trials was used as IIRV to measure individuals’ attention functions.

We first tested the hypothesis that children with ADHD would exhibit abnormal dynamics of brain states compared to their TD peers. We evaluated this by examining occupancy rates and mean lifetimes of latent brain states between the two groups. Our second hypothesis was that methylphenidate would restore normal dynamics of brain states in children with ADHD by reducing the difference in occupancy rates of affected brain states between the two groups.

Next, we determined the effect of methylphenidate on the latent brain states in relation to changes in IIRV induced by medication. We hypothesized that medication-induced changes in brain state dynamics would underlie reduced behavioral instability.

Finally, we analyzed differences in functional connectivity patterns between children with ADHD and TD children, as well as the impact of methylphenidate on these patterns and their contribution to the medication effect on IIRV in children with ADHD. We hypothesized that aberrant latent brain states would be characterized by abnormal functional connectivity between SN, FPN and DMN, and that medication-induced changes in functional connectivity would contribute to reduced IIRV.

## Methods

### Participants and study design

This study involves re-analysis of a previous published dataset [14]. The study protocol was approved by the Ethics Committee of the University of Fukui, Japan (Assurance no. 20170005). All participants and their parent(s) provided written informed consent for participation in this study. This study is registered with the University Hospital Medical Information Network (UMIN000027533).

Thirty-four children with ADHD and 65 TD children were recruited at the University of Fukui Hospital, Japan. Figure S1 shows the study design (see Supplementary Methods for details). Children with ADHD were scanned twice, in a randomized placebo-controlled double-blind crossover design. The administration order was counterbalanced across participants to address potential test-retest issues. During the first visit, they were administered osmotic release oral system methylphenidate (OROS-MPH) (1.0 ± 0.1 mg/kg) or placebo (lactose) under double-blind conditions as in previous studies [14, 45]. Five to eight hours after administration, when the methylphenidate concentration in the blood is maximal [46], they underwent a resting-state functional MRI (fMRI) scan and performed a standardized continuous performance task outside the MRI scanner.

During the second visit, within 1 to 6 weeks after the first visit, children with ADHD underwent a resting-state fMRI scan and performed the continuous performance task (CPT) after they took the second medicine: children who took OROS-MPH at the first visit took the placebo at the second visit under double-blind randomly assigned conditions, and vice versa.

TD children completed the same resting-state fMRI scan once without either OROS-MPH or placebo. Inclusion criteria for both groups included no contraindications for magnetic resonance imaging (MRI), full-scale intelligence quotient (FSIQ) > 70 (to exclude participants with intellectual disability), no history of severe head trauma or neurological abnormalities (e.g. epilepsy, arachnoid cysts). To minimize the potential impact of sex differences, we included only male participants, consistent with previous ADHD imaging studies [47, 48]. Participants with excessive head motion (translational movement greater than 3.0 mm, rotational movement greater than 3.0 degrees, and mean framewise displacement greater than 0.3 mm) during the scanning were excluded [47]. Seven children with ADHD were excluded because of refusal to participate, arachnoid cyst, and motion during the MRI, while 16 TD controls were excluded because of psychiatric disorders, and neurological abnormalities, leading to a final sample of 27 children with ADHD (age: 10.6 ± 1.8 years, range 7.3–15.5 years) and 49 TD controls (age: 11.1 ± 2.3 years, range 6.1–15.6 years). Nine patients with ADHD had autism spectrum disorder, 6 ADHD patients had oppositional defiant disorder, 2 had specific learning disorder, and 1 had developmental coordination disorder as comorbid disorders. While one of the patients with ADHD was medication-naïve, 25 were medicated with OROS-MPH (medication period was 22.2 ± 15.3 months, range 1–58 months), three with atomoxetine, and two with aripiprazole. Children with ADHD took their regularly prescribed medications between the two visits, but all participants were medication-free prior to MRI for at least 5 times half-life, including methylphenidate and atomoxetine, consistent with protocols from previous studies [41, 47]. See Supplementary Methods, Figure S1, and Table S1 for more details.

### Assessment of attention and cognitive control

A standardized CPT was administered to children with ADHD outside the MRI scanner under both methylphenidate and placebo conditions. The task consisted of a Go/NoGo paradigm in which children were presented with either a target or non-target stimulus on the screen for 100 msec, once every 2 s for 15 min across three 5-minute blocks. The target stimulus was a triangle, while the non-target stimulus was either a circle or a square. Children were required to press a button when a target stimulus was presented, and withhold response to non-targets. The test performance has been normalized to age-adjusted T-scores. IIRV was quantified using reaction time (RT) and standard deviation (RTSD) from correct Go trials. We examined medication-induced performance differences using paired *t*-tests.

### fMRI data acquisition

Functional images were acquired in a 3-T scanner (Discovery MR 750; General Electric Medical Systems, Milwaukee, WI) and a 32-channel head coil with a T2*-weighted gradient-echo echo-planar imaging sequence (TR = 2300 ms, TE = 30 ms, FA = 81°, FOV = 192 × 192 mm, matrix size = 64 × 64, voxel size = 3 × 3 mm). In total, 201 volumes were acquired for a total scanning time of 7 min 42 s. The participants were instructed to stay awake with eyes closed. Head movement was minimized by the placement of memory foam pillows around their head.

### fMRI data pre-processing

Resting state fMRI data were analyzed using SPM12 (http://www.fil.ion.ucl.ac.uk/spm) and DPARSF [49] with the following steps. First, the initial 10 volumes were discarded, and slice-timing correction was performed. The signal from each slice was realigned temporally to that obtained from the middle slice using sinc interpolation, followed by spatial realignment of 191 volumes to the mean volume. The re-sliced volumes were normalized to the Montreal Neurological Institute (MNI) space with a voxel size of 2 × 2 × 2 mm using the EPI template provided by SPM12. The normalized images were spatially smoothed with a 6-mm Gaussian kernel. Next, the non-neural noise in the time series was controlled, and several sources of spurious variance were removed from the data through linear regression.

### Region of interest (ROI) and time series

Eleven ROIs were determined from a previous study of attention and cognitive control [13], including bilateral anterior insula (AI), bilateral middle frontal gyrus (MFG), bilateral frontal eye field (FEF), bilateral intraparietal sulcus (IPS), dorsomedial prefrontal cortex (DMPFC), ventromedial prefrontal cortex (VMPFC), and posterior cingulate cortex (PCC). Each ROI was a 6-mm radius sphere centered at the peak voxel.

Time series of the 1^st^ eigenvalue was extracted from each ROI per subject. A multiple linear regression approach with 6 realignment parameters (3 translations and 3 rotations) was applied to each time series to reduce head-motion-related artifacts; the resulting time series was further band-pass filtered (0.008 Hz>*f* > 0.1 Hz).

### Bayesian switching dynamical systems (BSDS) model

We used a BSDS model [13] to uncover latent brain states during cognitive performance of the simple choice response task. A brief explanation of the BSDS model is in the Supplementary Method. Detailed theoretical derivations are provided in our previous study [13].

ROI timeseries from typically developing (TD) children, children with ADHD under placebo (Placebo) and methylphenidate (MPH) conditions were grouped together and analyzed in a common BSDS model. This yielded latent states common to all three groups. Key measures extracted from BSDS include occupancy rate and mean lifetime of latent brain state as well as functional connectivity of states. Two sample *t*-tests were then conducted to determine differences in occupancy rate and mean lifetime between TD children and children with ADHD in placebo condition. A threshold of *p* < 0.05 with family-wise error (FWE) correction was used to determine statistical significance in multiple comparisons. For brain state features that showed significant between-group differences, we specifically tested whether (i) methylphenidate reduced aberrancies in latent brain states using a paired *t*-test between methylphenidate and placebo conditions in children with ADHD, and (ii) methylphenidate normalized aberrancies in latent brain state to the level of TD children using two-sample *t*-tests between TD children and children with ADHD in methylphenidate condition. Statistical significance was determined at *p* < 0.05.

### Brain state dynamics in relation to behavioral performance

To understand the relationship between the latent dynamic brain state and out-of-scanner behavioral performance, we used multiple linear regression models to examine whether occupancy rates and mean lifetime of each brain is related to behavioral performance. Other potential confounds such as age, social-economic status (SES) and IQ were also included in the model. Statistical significance was determined at *p* < 0.05.

### Functional connectivity of time-varying latent brain states

To determine which dynamic functional connections are important for distinguishing different brain states, we conducted paired t-tests to examine medication effect on the covariance matrix between latent brain states derived from BSDS analysis and two-sample t-tests to examine group differences. The results were thresholded at *p* < 0.05 with false discovery rate (FDR) correction for multiple comparisons.

### Medication effect on functional connectivity in relation to its effect on IIRV

To determine whether medication effect on functional connectivity between the SN, FPN and DMN contributes to its effect on sustained attention, we first conducted *Pearson’s* correlation between the difference of methylphenidate versus placebo in function connectivity of the aberrant latent brain state and its difference in IIRV. The results were thresholded at *p* < 0.05 with FDRcorrection for multiple comparisons We conducted multiple linear regression analysis to examine the relation between methylphenidate-induced changes in functional connectivity and medication effects on IIRV, while controlling for potential confounds, such as age, IQ and SES. Statistical significance was determined at *p* < 0.05.

## Results

### Participants and demographic information

Ninety-nine children, including 34 boys with a clinical diagnosis of ADHD and 65 TD boys (6–15 years old) completed the study. Children with ADHD were enrolled in a double-blind, placebo-controlled crossover design as described previously (39–41). Children with ADHD were scanned twice using resting-state functional MRI under methylphenidate and placebo conditions, which were counterbalanced across participants. Each child with ADHD took osmotic release oral system methylphenidate (OROS-MPH) (1.0 ± 0.1 mg/kg) in the methylphenidate condition and lactose in the placebo condition. For ethical reasons, TD children were scanned once without administration of methylphenidate or placebo. Participants with excessive head motion during the scanning, psychiatric comorbidity or neurological abnormalities were excluded (see Methods and Supplementary Methods for details), leading to a final sample of 27 children with ADHD and 49 TD children.

The two groups did not differ in age and handedness (all *ps* > 0.05, two-sample t-test, Table S1). Children with ADHD had significantly higher inattention and hyperactivity/impulsivity scores than TD children (all *ps* < 0.001, two-sample *t*-test, Table S1). There was no difference in head motion (mean framewise displacement) between TD (0.075 ± 0.033 mm) and children with ADHD in the placebo condition (0.082 ± 0.041 mm) (*p* = 0.45). Head motion in children with ADHD in the MPH condition (0.058 ± 0.014 mm) was significantly lower than those in placebo condition (*p* < 0.001) and in TD (*p* < 0.002).

### Methylphenidate effects on sustained attention

Children with ADHD completed a standardized continuous performance task [50] outside the scanner in both methylphenidate and placebo conditions. The task consisted of target and non-target stimuli and children were required to press a button when a target was presented, and withhold response to non-targets. IIRV was used to assess individual’s sustained attention ability and the effect of methylphenidate. We found that methylphenidate administration significantly reduced IIRV in children with ADHD (*p* < 0.001).

### Aberrant latent brain state dynamics in children with ADHD

Latent state variables play a crucial role in characterizing the dynamics of brain activity. We used BSDS to probe latent state dynamics of a cognitive control system comprising key nodes of the SN, FPN, and DMN: bilateral anterior insula, middle frontal gyrus, frontal eye fields, inferior parietal lobe, posterior cingulate cortex (PCC), ventromedial prefrontal cortex (VMPFC), and pre-supplementary motor area (or dorsomedial prefrontal cortex, DMPFC) (Fig. 1B). These ROIs were determined using an independent study demonstrating strong cognitive control and attentional load effects in each of these regions [13].

BSDS uncovered 5 distinct latent brain states labeled S1, S2, S3, S4 and S5 in a combined group of children with ADHD under placebo condition and TD controls (Fig. 1C). Occupancy rate provides an indication of how frequently a specific latent brain state occurs, reflecting the likelihood of the brain being in that particular state at any given moment. It quantifies the proportion of time the brain spends in a specific state relative to other states. On the other hand, mean lifetime measures the duration of a latent brain state, offering insights into how long the brain typically remains in that state before transitioning to other states. Together, these variables provide valuable information about the temporal characteristics and prevalence of different latent brain states.

**Fig. 2:**
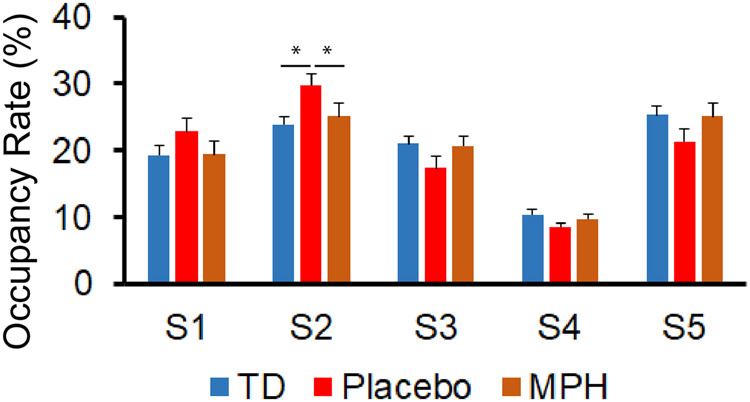
Methylphenidate normalizes aberrant latent brain states. Occupancy rate (OR) of the latent brain state S2 was significantly higher in children with ADHD than TD children (*p* < 0.05, FDR corrected) and methylphenidate significantly reduced hyper-OR of the latent brain state S2 in children with ADHD (*p* < 0.05).

We first examined group differences on occupancy rate across all the latent brain states. A two-way analysis of variance (ANOVA) with factors group (TD, ADHD) and state (S1, S2, S3, S4 and S5) (Fig. 1C) revealed a significant interaction between group and state (*F*_4,365_ = 5.89, *p* = 0.0001, Fig. 2), but no significant main effect of group and state (*ps* > 0.1). Post-hoc two-sample *t*-tests further revealed that the occupancy rate of S2 was significantly different between children with ADHD under placebo and TD children (*t*_48.7_ = 3.0, *p* = 0.004, FWE corrected *p* < 0.05, Fig. 2) with children with ADHD having increased occupancy rate of S2 than TD children. Occupancy rates of the four other latent brain states were not different between the two groups.

We conducted a similar ANOVA analysis to examine group differences on mean lifetimes across all the latent brain states but did not find significant interaction and main effects of group and state (all *ps* > 0.1).

These results identify a specific aberrancy in latent brain states associated with cognitive control systems in children with ADHD.

### Methylphenidate normalizes aberrant state dynamics in children with ADHD

Next, we tested whether methylphenidate could normalize abnormal state dynamics in children with ADHD. A two-way ANOVA with factors medication (placebo, methylphenidate) and state (S1, S2, S3, S4 and S5) revealed a significant main effect of state (*F*_4,250_ = 5.31, *p* = 0.0004, Fig. 2), but no significant main effect of group or interaction (*ps* > 0.9).

Because the occupancy rate of state S2 was significantly different between children with ADHD under placebo and TD children, we further specifically tested whether medication normalizes the occupancy rate of S2 in children with ADHD. A pairwise *t*-test showed that the occupancy rate of S2 was significantly different between placebo and methylphenidate condition in children with ADHD (*t*_26_ = 2.61, *p* = 0.01, Fig. 2), such that methylphenidate reduced the occupancy rate of S2 in children with ADHD. A follow-up two sample *t-*test further confirmed that there is no significant difference in the occupancy rate of S2 between children with ADHD in methylphenidate session and TD children (*p* > 0.5).

These results suggest that methylphenidate administration reduces aberrant latent brain states associated with cognitive control systems in children with ADHD, normalizing them to levels observed in TD children.

### Relationship between methylphenidate-induced changes in brain state dynamics and response variability

We then determined whether methylphenidate-induced changes in brain state dynamics are associated with changes in IIRV in the CPT. We focused on state S2 which we found to be aberrant in children with ADHD, as detailed above. Specifically, we conducted a multiple linear regression analysis to evaluate the relationship between methylphenidate-induced changes in occupancy rate and mean lifetimes of S2 and medication induced changes in IIRV controlling for potential confounding influences of age, IQ and SES. Methylphenidate-induced changes in occupancy rate of S2 is the only significant predictor for medication-induced changes in IIRV (*p* = 0.047, Supplementary Table S2). Control analyses of the dynamic properties of other latent brain states (S1, S3, S4 and S5) are not associated with methylphenidate-induced changes in IIRV (all *ps* > 0.05). These results show that methylphenidate-induced reductions in aberrant latent brain states are associated with behavioral improvements in ADHD.

### Hyper-connectivity of the DMN in aberrant brain state in children with ADHD

**Fig. 3:**
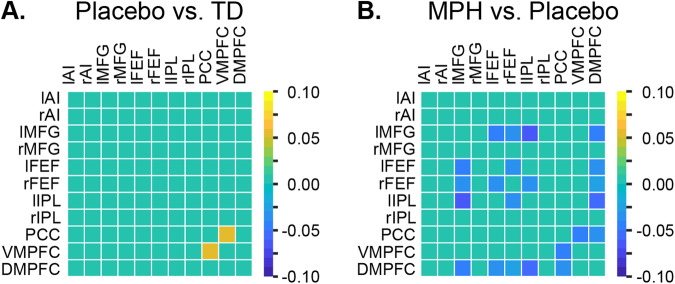
Methylphenidate reduces aberrant state-specific hyper-connectivity. Children with ADHD under placebo condition showed (**A**) hyper-connectivity between PCC and VMPFC in the state S2 than TD children (*p* < 0.05, FDR corrected) and **B** methylphenidate reduced hyper-connectivity between the DMN nodes as well as connectivity between SN, FPN and DMN nodes in the state S2(*p* < 0.05, FDR corrected). State S2 was the latent brain state with higher occupancy rate in children with ADHD, compared to controls. lAI left anterior insula, rAI right anterior insula, DMPFC Dorsomedial prefrontal cortex, lMFG left middle frontal gyrus, rMFG right middle frontal gyrus, lFEF left frontal eye field, rFEF right frontal eye field, lIPL left intraparietal lobule, rIPL right intraparietal lobule, PCC Posterior cingulate cortex, VMPFC Ventromedial prefrontal cortex.

We sought to determine differences in functional connectivity associated with S2, the aberrant latent brain states in children with ADHD. Here we leveraged the ability of BSDS to identify connectivity patterns associated with each brain state in each individual. Compared to TD children, children with ADHD under placebo showed stronger functional connectivity between the PCC and VMPFC nodes of the DMN (*p* < 0.05, FDR corrected, Fig. 3). This result reveals that hyper-connectivity of DMN contributes to aberrant latent brain states in children with ADHD. Control analysis revealed that functional connectivity between the PCC and VMFPC in other brain states were not different between children with ADHD under placebo and TD children (all *ps* > 0.05).

### Methylphenidate influences functional connectivity of aberrant brain state in children with ADHD

Next, we examined the effect of methylphenidate on functional connectivity in the aberrant brain state, S2, in children with ADHD. We found that the medication reduces hyper-connectivity between PCC and VMPFC in children with ADHD (*p* < 0.05, FDR corrected, Fig. 3). Additionally, methylphenidate reduced intra-network functional connectivity associated with multiple nodes of the DMN, SN, and FPN (*p* < 0.05, FDR corrected, Fig. 3). There were no differences in functional connectivity of state S2 between TD children and children with ADHD in methylphenidate condition (*p* > 0.05). These results demonstrate that methylphenidate normalizes abnormal latent brain state functional connectivity of the DMN in children with ADHD.

### Methylphenidate modulation on SN-DMN functional connectivity predicts its effect on IIRV

**Fig. 4:**
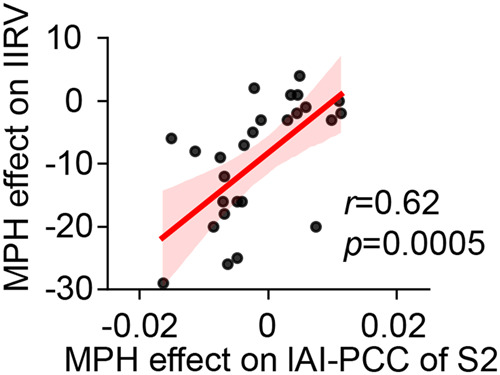
State-specific effects of methylphenidate on brain and behavioral dynamics. Methylphenidate effect on the functional connectivity of lAI and PCC of the latent brain state S2 was significantly associated with the medication effect on the IIRV (*r* = 0.62, *p* = 0.0005). State S2 was the latent brain state with higher occupancy rate in children with ADHD, compared to controls. lAI left anterior insula, rAI; PCC Posterior cingulate cortex.

Finally, we examined whether methylphenidate-induced changes in functional connectivity of the aberrant brain state, S2, are associated with medication-induced changes in IIRV. We found that medication-induced changes in functional connectivity between left AI node of the SN and PCC node of the DMN in state S2 is significantly correlated with medication-induced changes in IIRV in children with ADHD (*r* = 0.62, *p* = 0.0005, *Pearson*’s correlation, *p* < 0.05 FDR corrected, Fig. 4). This relationship held after controlling age, IQ and SES education level (*p* = 0.0001, Supplementary Table S3). These results suggest that changes in cross-network interactions between the SN and DMN contribute to behavioral improvements associated with methylphenidate. Control analysis found no significant correlation between medication-induced functional connectivity changes in any other brain states and changes in IIRV (all *ps* > 0.05).

### Robustness of findings

We conducted additional analyses to determine the robustness of our findings. Specifically, we examined whether medication effects on brain-behavior association are stable with respect to head motion, exclusion of IQ as covariates in the regression model. We also examined whether results were influenced by comorbidity with autism in children with ADHD. Results from these analyses replicated our main findings, highlighting the robustness of our findings (Supplementary Materials).

## Discussion

Methylphenidate is a commonly used stimulant for treating inattention symptoms in children with ADHD. We used a novel state-space approach to investigate aberrancies in latent brain states associated with dynamics of cognitive control networks in childhood ADHD and then determined whether methylphenidate normalizes these aberrancies and improves behavioral performance on a sustained attention task. Our analysis revealed aberrant brain state dynamics, which were remediated by methylphenidate and correlated with improved attention. Methylphenidate also improved brain state-related functional connectivity and remediated hyper-connectivity in the DMN. Additionally, medication-induced changes in cross-network functional connectivity between the DMN and SN predicted medication effects on sustained attention. Our findings demonstrate that methylphenidate remediates attention problems in children with ADHD by normalizing aberrant dynamic brain mechanisms, thus advancing our understanding of the brain mechanisms associated with the effect of methylphenidate on attention.

### Methylphenidate normalizes aberrant brain state dynamics in children with ADHD

One prominent neurocognitive model proposes that higher levels of attentional fluctuations in individuals with ADHD may arise from abnormal brain dynamics [20]. Consistent with this model, prior research has demonstrated that children with ADHD exhibit atypical time-varying brain network interactions in comparison to TD children [14, 19]. However, these findings were limited by the methodology used as it relies on ad hoc procedures for determining critical parameters in sliding window and temporal clustering approach [44], as well as difficulties in determining the optimal number of spatial and temporal components in temporal ICA.

The current study overcomes these limitations by using a novel BSDS algorithm to uncover latent brain states characterized by unique spatiotemporal circuit properties. BSDS identified 5 latent brain states, characterized by distinct patterns of intrinsic functional connectivity between key nodes of the SN, FPN and DMN. Among these states, state S2 was found to have a significantly higher occupancy rate in children with ADHD than TD children, indicating abnormal brain dynamics during resting state. Our previous study found differences in brain connectivity between children with ADHD and their TD peers [51]. However, in this study, we went a step further and identified a specific abnormal latent brain state that was characterized by a unique pattern of functional connectivity and elevated occupancy rate in children with ADHD. This discovery opens up the possibility of using the generative framework of the BSDS model to develop biomarkers on this unique latent brain state for the diagnosis of ADHD or attention problem, thus offering a significant clinical potential. Crucially, methylphenidate decreased the hyper-occupancy rate of this latent brain state S2, bringing it more in line with TD children. This finding suggests that methylphenidate can restore aberrant latent brain state dynamics in children with ADHD.

Moreover, changes in the occupancy rate of state S2 induced by methylphenidate were found to predict medication’s effect on sustained attention, as measured by the IIRV in a continuous performance task. While previous studies have shown that methylphenidate can lower IIRV in children with ADHD [15], the dynamic brain mechanism through which methylphenidate reduces behavioral instability was not known. Our findings suggest that improving brain state dynamics, specifically reducing the occupancy rate of state S2, is key to improving sustained attention function in children with ADHD. This is also consistent with a recent finding showing that occupancy rates of latent brain states during cognitive tasks are correlated with IIRV [11]. Moreover, this relationship was not found in other latent brain states, suggesting that state S2 is uniquely impacted by the single dosage treatment of methylphenidate.

Together, our findings suggest that a single dose of methylphenidate can remediate aberrant latent brain state dynamics of the cognitive control system in children with ADHD, and, furthermore, changes in brain dynamics induced by methylphenidate are associated with improved sustained attention.

### Methylphenidate normalizes state-specific hyper-connectivity of the DMN in children with ADHD

Our next goal was to uncover latent space features that define the abnormal state S2 in children with ADHD and determine whether methylphenidate can normalize these features. Each latent brain state is characterized by distinct patterns of functional connectivity between the key nodes of the SN, FPN and DMN. These networks play an essential role in a wide range of attention and cognitive control tasks, with a high degree of reproducibility [52–54]. Previous studies have revealed abnormal task-evoked activation in the SN and FPN [38, 55–59] and less deactivation and more variable activity in the DMN [57, 60, 61] during attention and cognitive control tasks as well as aberrant intrinsic connectivity among the SN, FPN and DMN in children with ADHD [14, 19, 39, 41]. However, the precise mechanisms through which methylphenidate modulates dynamic connectivity between these networks have not been clear.

Our findings provide novel evidence on aberrant functional connectivity of cognitive control systems in children with ADHD and the impact of methylphenidate on these systems. We found that, compared to controls, children with ADHD exhibited higher functional connectivity between the two key nodes of the DMN, the posterior cingulate cortex (PCC) and the ventromedial prefrontal cortex (vmPFC). This is in line with previous findings that show ADHD-related dysfunction in the DMN [62]. Furthermore, we demonstrated that methylphenidate reduces hyper-connectivity between PCC and vmPFC in children with ADHD. Our study provides strong evidence that methylphenidate normalizes abnormal functional connectivity of the DMN in children with ADHD, a brain system that is closely linked to attention function and inattention symptom [39, 41, 42].

### Methylphenidate’s modulation on SN-DMN connectivity contributes to its effect on attention

Our last goal was to determine whether changes in functional connectivity induced by methylphenidate in the abnormal state S2 were linked to medication’s impact on sustained attention. We used IIRV from a sustained attention task as high response variability is a hallmark of inattention. We found that methylphenidate’s impact on functional connectivity between left AI and PCC in state S2 was associated with its effect on IIRV in children with ADHD. This aligns with the triple-network model of attention and cognitive control [63].

The triple-network model suggests that the SN plays a critical role in facilitating access to attention and cognitive control resources and modulating its interaction with the FPN and DMN, which are involved in externally-oriented attention and internally-oriented mental processes. Studies in neurotypical adults have shown that attention and cognitive control modulates interaction between key nodes in the SN (e.g. AI) and DMN (e.g. PCC) [64, 65] and poor attention and cognitive control functions are accompanied by disrupted interaction between AI and PCC in patients with traumatic brain injury [66]. Intrinsic connectivity between DMN regions and SN regions has also been found to be correlated with attention problems [39, 40, 67]. Our finding goes beyond prior research by establishing that methylphenidate’s impact on the functional connectivity between the AI and PCC drives its effect on sustained attention in a specific latent brain state S2. This also converges with our recent work showing that functional connectivity between AI and PCC is correlated with inattention scores in children during cognitive performance [11]. These results highlight the crucial role of the AI-PCC connection in attention function, which may be the crucial brain mechanism through which methylphenidate improves inattention symptoms in children with ADHD. Notably, this behaviorally relevant medication effect on the AI-PCC connection was observed only with state S2. Our findings suggest that the effects of methylphenidate on sustained attention are dependent on the brain state. This state specificity provides new insights into how methylphenidate modulates sustained attention and cognitive control in individuals with ADHD, and highlights the importance of considering the dynamic nature of brain activity in the context of medication effects.

### Limitations

We acknowledge several limitations in this study. Our study participants were all male, not all drug naïve, and spanned a wide range from 6 to 15. To control for the effects of psychostimulants, all participants were medication-free for at least 5 half-lives prior to the MRI scans. For ethical reasons, participants were allowed to take medication between study days. Our randomized placebo-controlled double-blind crossover design involved similar washout protocols for the methylphenidate and placebo groups, thus controlling for the effects of use of medication between study days. However, it is still possible that the effect of medication may be influenced by administration procedures, the length of washout and medication dosage. Additionally, it is unknown how the diverse neurodevelopmental stages across participants impact the findings [68]. Further studies with larger samples of drug naïve males and females with ADHD are needed to determine how medication history, sex, and development stage along with other medication factors can impact the effects of methylphenidate on cognitive control circuits. While we used a randomized controlled design for children with ADHD, TD controls were only studied at baseline for ethical reasons. Finally, designs that incorporate methylphenidate and placebo arms in TD controls, with multiple measures of behavioral and clinical measures associated with ADHD, may provide further insights into how methylphenidate impacts cognitive control circuits and sustained attention.

## Conclusion

Our study sheds new light on the mechanisms by which methylphenidate improves attention and cognitive control in children with ADHD. Using a Bayesian dynamic systems model, we investigated how methylphenidate modulates the dynamics of latent brain states and how this modulation affects behavioral variability. We found that children with ADHD exhibited aberrant latent brain state dynamics compared to typically developing children, with a single latent state showing particularly abnormal dynamics. Notably, methylphenidate was found to normalize the abnormal dynamics of this latent brain state, which was characterized by hyper-connectivity in the default mode network. Furthermore, our analysis revealed that methylphenidate-induced changes in state-specific functional interactions between the salience and default mode networks were associated with improved sustained attention. These findings provide new insights into the impact of methylphenidate on dynamic brain states and its ability to mitigate behavioral instability and inattention in children with ADHD. By revealing the circuit-level and state-specific mechanisms underlying the therapeutic effects of methylphenidate, our findings have the potential to inform the development of more effective treatments for ADHD. More generally, our Bayesian dynamical systems models may be of value to clinicians and researchers investigating the neural mechanisms underlying pharmacological treatments for a wide range of psychiatric disorders.

### Supplementary information

Supplementary Material

## Data Availability

All code will be shared on SCSNL GitHub upon publication.
