# Supplementary material for: Bayesian dynamical system analysis of the effects of methylphenidate in children with attention-deficit/hyperactivity disorder: a randomized trial
Source: Neuropsychopharmacology. 2023 Jul 25;48(11):1690–8. doi: 10.1038/s41386-023-01668-3 (PMC10516959; doi:10.1038/s41386-023-01668-3)
Supplement: Supplementary file 1 — Supplementary Material [file 41386_2023_1668_MOESM1_ESM.pdf]

Methylphenidate normalizes abnormal state dynamics of cognitive control system in children with attention-deficit/hyperactivity disorder: a randomized trial

Weidong Cai, Yoshifumi Mizuno, Akemi Tomoda, Vinod Menon

Supplemental Methods

Study design and participants

The overall design of the study is shown in **Figure S1**. In total, we recruited 34 children with attention-deficit/hyperactivity disorder (ADHD) and 65 typically developing (TD) children at the University of Fukui Hospital, Japan, and the neighboring community. The diagnosis of ADHD was based on the Diagnostic and Statistical Manual of Mental Disorders, Fifth Edition (DSM-5) [1], and was confirmed in structured interviews using the ADHD module of the Japanese Version of the Kiddie Schedule for Affective Disorders and Schizophrenia for School-Aged Children-Present and Lifetime Version (K-SADS-PL-J) [2,3]. The TD group had no family history of psychiatric diseases. Intellectual capacities were estimated via the Wechsler Intelligence Scale for Children-Fourth (WISC-IV) [4]. Parents of children in both groups were asked to complete Conners 3rd Edition (Conners) instrument [5] to evaluate inattention, and hyperactivity/impulsivity symptoms under no medication. Handedness was assessed using the Edinburgh Handedness Inventory [6].

Thirty-four children with ADHD were randomly assigned to methylphenidate or placebo conditions. One child with ADHD declined to participate. The study medicine manager assigned the study drug to participants using block randomization. Each time a participant was enrolled, the study drug was assigned according to the label of the study drug which was recorded in a random order in advance. During the first visit, participants either took osmotic release oral system methylphenidate (OROS-MPH; 1.0mg/kg: 1.0 ± 0.1mg/kg) [7] or a placebo (lactose) under double-blind conditions. Therefore, neither the participants (both children and parents) nor the examiners know whether they took OROS-MPH or placebo. 5-8 hours after administration of the placebo or methylphenidate [8], when methylphenidate concentration in the blood is maximal [9], participants underwent a resting-state functional MRI (fMRI) scan. Children with ADHD were also administered a continuous performance task [10] outside the MRI scanner. Of the 17 children with ADHD who received methylphenidate, 15 completed both MRI and CPT, and 2 were excluded because they did not stay still during the MRI scan. Of 16 children with ADHD who received the placebo, 15 completed MRI and CPT, and 1 was excluded because an arachnoid cyst was detected.

At the second visit, within 1 to 6 weeks from the first visit (mean 17.9 days, standard deviation 9.6 days), they underwent a second resting-state fMRI and performed the CPT again following administration of either the placebo or methylphenidate under double-blind conditions. Children with ADHD who took OROS-MPH at the first visit now took the placebo at the second visit, and vice versa. All 15 children with ADHD who received methylphenidate completed MRI and CPT, and of the 15 children with ADHD who received the placebo, 12 completed MRI and CPT, and 3 were excluded because 1 declined MRI, and 2 did not stay still during the MRI scan. Of 65 TD children, 16 were excluded, as 6 were female, 6 had psychiatric disorders, and 4 had neurological abnormalities. TD controls were scanned once without OROS-MPH or placebo.

Data analyses involved data from 76 subjects, comprising 27 patients with ADHD and 49 TD controls. 14 children with ADHD were classified as combined presentation, and 13 were predominantly inattentive presentation. 9 patients with ADHD had autism spectrum disorder, 6 ADHD patients had oppositional defiant disorder, 2 had specific learning

disorder, and 1 had developmental coordination disorder as comorbid disorders. While one of the patients with ADHD was medication-naïve, 25 were medicated with OROS-MPH, three with atomoxetine, and two with aripiprazole. All participants were medication-free prior to MRI for at least 5 times half-lives, including methylphenidate and atomoxetine, consistent with protocol from previous studies. Of the 25 patients who had taken OROS-MPH orally, 2 had stopped taking it for more than 1 month and 23 had stopped taking it for more than 48 hours. Mean FD in the ADHD-MPH (0.058±0.014mm) was significantly lower than the ADHD-Placebo and TD groups (*ps* < 0.001, = 0.002). There were no differences in mean FD between the ADHD-Placebo (0.082±0.041mm) and TD groups (0.075±0.033mm) (*p* = 0.450).

Adverse events on methylphenidate condition were 2 loss of appetite, 2 insomnia, 1 abdominal pain, and 1 fatigue, all of which were temporal and mild. No serious adverse events were observed.

BSDS generative model

Bayesian switching dynamical systems (BSDS) is a powerful state-space generative model for uncovering latent brain state dynamics [11]. BSDS identifies brain states and their dynamic spatiotemporal properties in an optimal latent subspace. These properties allowed us to identify shared brain states across multiple cognitive tasks.

Here we briefly describe the BSDS model [11]. Let $\boldsymbol{y}_t^s$ denote a D-dimensional vector of observed fMRI measurements in time t and for subject s. Further, let $\boldsymbol{z}_t^s$ denote a 1-of-K discrete vector of latent state variables of a hidden Markov model (HMM) with elements $z_{kt}^s, \forall k = 1, \dots, K$. Two consecutive time instances are dependent via a first-order Markov chain through an HMM. Specifically, probability distribution of $\boldsymbol{z}_t^s$ depends on the state of the previous latent variable $\boldsymbol{z}_{t-1}^s$ through a conditional distribution $p(\boldsymbol{z}_t^s \mid \boldsymbol{z}_{t-1}^s, \boldsymbol{A}) = \prod_{k=1}^K \prod_{j=1}^K A_{jk}^{z_{t-1,j}^s z_{tk}^s}$ for all $t > 1$ represented by the transition probabilities $\boldsymbol{A}$, where $A_{jk} \equiv p\left(z_{tk}^s = 1 \mid z_{t-1,j}^s = 1\right)$, and a marginal distribution $p(\boldsymbol{z}_1^s \mid \boldsymbol{\pi}) = \prod_{k=1}^K \pi_k^{z_{1k}^s}$ represented by a vector of initial probabilities $\boldsymbol{\pi}$ where $\pi_k \equiv p(z_{1k}^s = 1)$ [12]. Next, we assume that at a given mode of the system given by the latent state $z_{kt}^s = 1$, observed vector $\boldsymbol{y}_t^s$ is generated via a state space model in form of

Equations 1-2:

$$\boldsymbol{y}_t^s = \boldsymbol{U}_k \boldsymbol{x}_{kt}^s + \boldsymbol{\mu}_k + e_{kt}, \qquad \forall t \mid z_{kt}^s = 1, \tag{1}$$

$$\boldsymbol{x}_{kt}^s = \overline{\boldsymbol{X}}_{kt}^s \vec{\boldsymbol{V}}_k + \boldsymbol{\epsilon}_{kt}, \qquad \forall t \mid z_{kt}^s = 1. \tag{2}$$

Line 1 of the model represents a probabilistic factor analysis model [13,14] where $\boldsymbol{U}_k$ is a $D \times P$ dimensional linear transformation matrix that transforms data to a subspace of lower dimensionality, $P < D$, described using a P-dimensional vector of latent space variables $\boldsymbol{x}_{kt}^s$ mediated by an overall bias $\boldsymbol{\mu}_k$ and a measurement noise $\boldsymbol{e}_{kt} \sim \mathcal{N}(\boldsymbol{0}, \boldsymbol{\Psi}_k)$. Line 2 represents an autoregressive (AR) process of order R defined on the latent space variables of the factor analysis model [15]. $\vec{\boldsymbol{V}}_k$ is a vector of AR coefficients. $\overline{\boldsymbol{X}}_{kt}^s =$

$\text{diag}(\overline{\boldsymbol{x}}^{s}_{kt})$ is a block diagonal isotropic matrix with elements of $\overline{\boldsymbol{x}}^{s}_{kt} = ({\boldsymbol{x}^{s}_{k,t-1}}^{\mathrm{T}}, {\boldsymbol{x}^{s}_{k,t-2}}^{\mathrm{T}}, \dots, {\boldsymbol{x}^{s}_{k,t-R}}^{\mathrm{T}})$ representing latent space variables from the previous R time frames where T indicates the transpose operator. $\boldsymbol{\epsilon}_{kt} \sim \mathcal{N}(\boldsymbol{m}_k, \boldsymbol{\Sigma}_k)$ models the remaining error term in latent space. An AR process of a first order, $R = 1$, is defined on the representations of the observations in the latent subspace, $\boldsymbol{x}^{s}_{kt}$. Variables $(\boldsymbol{\Psi}_{\boldsymbol{k}}, \vec{\boldsymbol{V}}_k, \boldsymbol{m}_k, \boldsymbol{\Sigma}_k)$ are global latent variables which are not a function of time t. Detailed theoretical derivations are provided in our previous study [11].

Supplemental Results

IIRV in association with accuracy

IIRV is significantly correlated with omission error and commission error in children with ADHD under Placebo ($r_{IIRV, OE}$=0.76, *p*=4.9e-06, $r_{IIRV, CE}$=0.76, *p*=5.2e-06) and Methylphenidate ($r_{IIRV, OE}$=0.60, *p*=0.0009, $r_{IIRV, CE}$=0.79, *p*=1.2e-06) conditions.

Robustness of main findings

Brain-behavior association with head motion as a covariate

To test whether brain-behavior association findings were influenced by head motion, we repeated multiple linear regression models with IQ as a covariate (**Supplementary Table S4&5**) and found equivalent results as our main findings.

Brain-behavior association without IQ as a covariate

To test whether using IQ as a covariate leads to biased findings, we repeated multiple linear regression models without IQ as a covariate (**Supplementary Table S6&7**), and found equivalent results as our main findings.

Brain state dynamics and medication effect in ADHD children without ASD comorbidity

After excluding ADHD children with ASD comorbidity, the new dataset included 18 children with ADHD and 49 TD children. BSDS revealed 4 distinct latent brain states labeled S1, S2, S3 and S4. Please note that the state numbers are randomly assigned, which are not mapped to the states in the original analyses.

We first examined group differences on occupancy rate across all the latent brain states. A two-way analysis of variance (ANOVA) with factors group (TD, ADHD) and state (S1, S2, S3 and S4) revealed a significant interaction between group and state ($F_{3,256}$=6.29, *p*=0.0004, **Supplementary Figure S2**), but no significant main effect of group and state (*ps*>0.2). Post-hoc two-sample *t*-tests further revealed that the occupancy rate of S1 was significantly different between children with ADHD under placebo and TD children (*t*=2.02, *p*=0.04, **Supplementary Figure S2**) with children with ADHD having increased occupancy rate of S2 than TD children.

Next, we tested whether methylphenidate could normalize abnormal state dynamics in children with ADHD. A two-way ANOVA with factors medication (placebo, methylphenidate) and state (S1, S2, S3 and S4) revealed a significant main effect of state ($F_{3,128}$=14.61, *p*=3E-08, **Supplementary Figure S2**), but no significant main effect of group or interaction (*ps*>0.1).

Because the occupancy rate of state S1 was significantly different between children with ADHD under placebo and TD children, we further specifically tested whether medication normalizes the occupancy rate of S1 in children with ADHD. A pairwise *t*-test showed that the occupancy rate of S1 was significantly different between placebo and methylphenidate condition in children with ADHD (*t*=2.35, *p*=0.03, **Supplementary Figure S2**), such that

methylphenidate reduced the occupancy rate of S1 in children with ADHD. A follow-up two sample *t*-test further confirmed that there is no significant difference in the occupancy rate of S1 between children with ADHD in methylphenidate session and TD children ($p>0.8$).

Finally, we examined whether methylphenidate-induced functional connectivity changes in the aberrant brain state, S1, are associated with changes in IIRV. We found that functional connectivity between left AI node of the SN and DMN nodes in state S1 is associated with IIRV in children with ADHD: lAI-PCC was marginally significantly correlated with IIRV ($r=0.43$, $p=0.07$, *Pearson*'s correlation); lAI-VMPFC was significantly correlated with IIRV ($r=0.57$, $p=0.01$, *Pearson*'s correlation); and rAI-VMPFC was significantly correlated with IIRV ($r=0.71$, $p=0.001$, *Pearson*'s correlation). All these relationships were significant after controlling age, IQ SES education level and head motion ($p<0.05$, **Supplementary Table S8**). These results suggest that changes in cross-network interactions between the SN and DMN contribute to behavioral improvements associated with methylphenidate.

Supplemental Figures

Supplementary Figure S1. Study design. Randomized placebo-controlled double-blind crossover design to investigate the brain circuit mechanisms that underlie the therapeutic effects of methylphenidate treatment in children with ADHD. CPT: continuous performance task; MRI: magnetic resonance imaging; TD: typically developing.

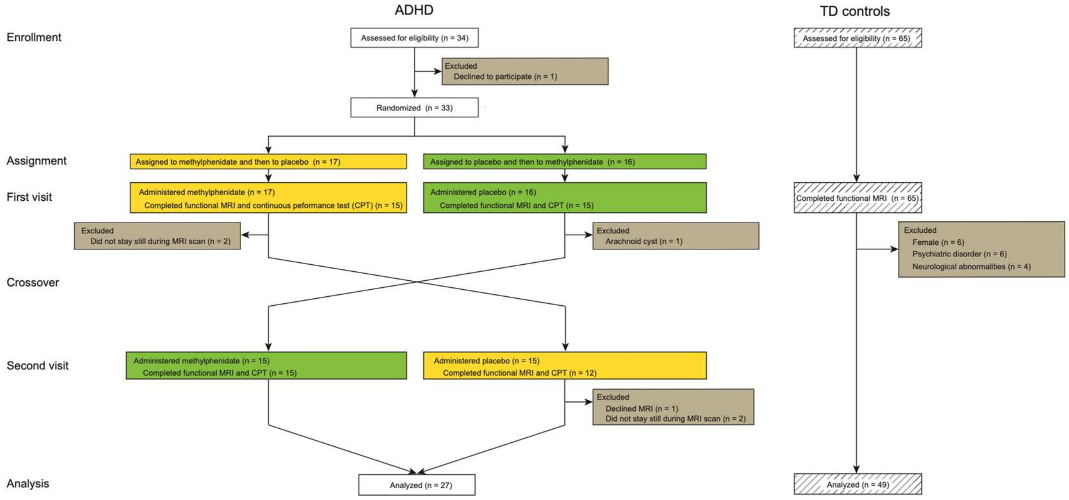

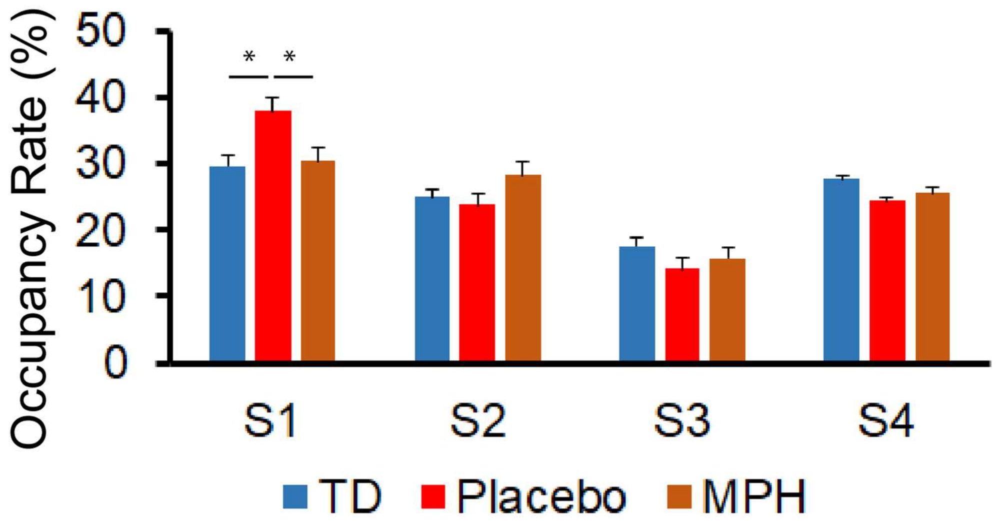

Supplementary Figure S2. Replication analysis (excluding ADHD children with comorbidity of ASD): Methylphenidate normalizes aberrant latent brain states. Occupancy rate (OR) of the latent brain state S1 was significantly higher in children with ADHD than TD children ($p<0.05$) and methylphenidate significantly reduced hyper-OR of the latent brain state S1 in children with ADHD ($p<0.05$).

Supplemental Table S1. Demographic and behavioral characteristics of participants included in data analysis.

	ADHD	TD	*p*
**Sample size**	27	49	-
**Age (years)**	10.6±1.8	11.1±2.3	0.397
**Handedness (R/L)**	25/2	47/2	0.534
**FSIQ**	90.8±8.7	105.2±11.0	<0.001***
**Conners IN (T)**	78.4±12.1	45.7±8.5	<0.001***
**Conners HY (T)**	73.1±15.3	42.9±3.9	<0.001***

ADHD: attention-deficit/hyperactivity disorder; TD: typically developing; R: right; L: left; FSIQ: full scale intelligence quotient; IN: inattention; HY: hyperactivity/impulsivity; ***$p < 0.001$

Supplemental Table S2. Multiple linear regression analysis showed that methylphenidate effect on occupancy rate (OR) of the latent state S2 is the robust predictor of the medication effect on IIRV after controlling Age, IQ and SES education level. The state S2 was the latent brain state with hyper-occupancy rate associated with the disorder. OR: occupancy rate; ML: mean lifetimes.

	betas	*t value*	*p value*
IIRV~OR(S2)+ML(S2)+Age+IQ+SES			
OR(S2)	47.18	2.11	0.04*
ML(S2)	-0.81	-0.73	0.47
Age	1.79	1.63	0.12
IQ	-0.16	-0.74	0.48
SES education level	1.64	1.08	0.29

*, $p<0.05$

Supplemental Table S3. Multiple linear regression analysis showed that methylphenidate effect on IAI-PCC connectivity of the latent state S2 is the robust predictor of the medication effect on IIRV after controlling Age, IQ and SES education level. The state S2 was the latent brain state with hyper-occupancy rate associated with the disorder.

	betas	*t value*	*p value*
IIRV~IAI-PCC+Age+IQ+SES			
IAI-PCC	94.08	4.72	0.0001***
Age	1.29	1.53	0.14
IQ	-0.04	-0.27	0.79
SES education level	2.9	2.46	0.02*

*, p<0.05; ***, p<0.001.

Supplemental Table S4. Replication analysis (including head motion as a covariate): Multiple linear regression analysis showed that methylphenidate effect on occupancy rate (OR) of the latent state S2 is the robust predictor of the medication effect on IIRV after controlling Age, IQ , SES education level and head motion. The state S2 was the latent brain state with hyper-occupancy rate associated with the disorder. OR: occupancy rate; ML: mean lifetimes; FD: frame-wise displacement

	betas	*t value*	*p value*
IIRV~OR(S2)+ML(S2)+Age+IQ+SES+FD			
OR(S2)	47.97	2.09	0.049*
ML(S2)	-0.83	-0.73	0.47
Age	1.85	1.63	0.12
IQ	-0.16	-0.79	0.44
SES	1.65	1.06	0.3
FD	14.5	0.38	0.71

Supplemental Table S5. Replication analysis (including head motion as a covariate): Multiple linear regression analysis showed that methylphenidate effect on IAI-PCC connectivity of the latent state S2 is the robust predictor of the medication effect on IIRV after controlling Age, IQ, SES education level and head motion. The state S2 was the latent brain state with hyper-occupancy rate associated with the disorder. OR: occupancy rate; ML: mean lifetimes; FD: frame-wise displacement.

medication effect	*betas*	*t value*	*p value*
IIRV~IAI-PCC+Age+IQ+SES+FD			
IAI-PCC	100.24	4.85	0.00008***
Age	1.13	1.32	0.2
IQ	0.002	0.01	0.99
SES	3.03	2.57	0.02*
FD	-31.55	-1.08	0.29

Supplementary Table S6. Replication analysis (excluding IQ as a covariate): Multiple linear regression analysis showed that methylphenidate effect on occupancy rate (OR) of the latent state S2 is the robust predictor of the medication effect on IIRV after controlling Age and SES education level. The state S2 was the latent brain state with hyper-occupancy rate associated with the disorder. OR: occupancy rate; ML: mean lifetimes.

	betas	*t value*	*p value*
IIRV~OR(S2)+ML(S2)+Age+SES			
OR(S2)	46.44	2.01	0.04*
ML(S2)	-86	-0.78	0.44
Age	1.67	1.55	0.14
SES	1.54	1.02	0.32

Supplementary Table S7. Replication analysis (excluding IQ as a covariate): Multiple linear regression analysis showed that methylphenidate effect on IAI-PCC connectivity of the latent state S2 is the robust predictor of the medication effect on IIRV after controlling Age and SES education level. The state S2 was the latent brain state with hyper-occupancy rate associated with the disorder.

medication effect	*betas*	*t value*	*p value*
IIRV~IAI-PCC+Age+SES			
IAI-PCC	94.74	4.89	0.00006***
Age	1.25	1.54	0.14
SES	2.9	2.5	0.02*

Supplementary Table S8. Replication analysis (excluding ADHD children with comorbidity of ASD)**:** Multiple linear regression analysis showed that methylphenidate effect on AI-DMN connectivity of the latent state S1 is the robust predictor of the medication effect on IIRV after controlling Age, IQ, SES education level and head motion. The state S1 was the latent brain state with hyper-occupancy rate associated with the disorder.

medication effect	*betas*	*t value*	*p value*
IIRV~lAI-PCC+Age+IQ+SES+FD			
lAI-PCC	26.78	2.21	0.04*
Age	3.49	2.87	0.01*
IQ	-0.29	-1.06	0.31
SES	1.75	1.33	0.21
FD	-2.15	-0.05	0.97

IIRV~lAI-VMPFC+Age+IQ+SES+FD			
lAI-VMPFC	53.35	4.21	0.001***
Age	3.95	4.28	0.001***
IQ	-0.11	-0.52	0.61
SES	1.22	1.24	0.24
FD	-37.88	-1.01	0.35

IIRV~rAI-VMPFC+Age+IQ+SES+FD			
rAI-VMPFC	60.61	2.94	0.01*
Age	2.46	2.11	0.06
IQ	-0.02	-0.06	0.95
SES	0.11	0.08	0.93
FD	-8.38	-0.19	0.85

Reference

1 Association AP. (American Psychiatric Association, Washington, DC, 2013).

2 Kaufman J, Birmaher B, Brent D, Rao U, Flynn C, Moreci P, et al. Schedule for Affective Disorders and Schizophrenia for School-Age Children Present and Lifetime version (K-SADS-PL): Initial reliability and validity data. J Am Acad Child Psy. 1997;36(7):980-88.

3 Miyawaki D, Suzuki F, Mamoto A, Takahashi K, Kiriike N. The reliability and validity of Japanese version of the schedule for affective disorders and schizophrenia for school-age children — present and lifetime version (K-SADS-PL). Japanese J Child Adolesc Psychiatry. 2003;197.

4 Wechsler D. Wechsler Intelligence Scale for Children-WISC-IV. Psychological Corporation: San Antonio, TX; 2003.

5 Conners CK. Conners 3rd edition. Multi-Health Systems: Toronto, Ontario, Canada; 2008.

6 Oldfield RC. The assessment and analysis of handedness: the Edinburgh inventory. Neuropsychologia. 1971;9(1):97-113.

7 Wilens T, McBurnett K, Stein M, Lerner M, Spencer T, Wolraich M. ADHD treatment with once-daily OROS methylphenidate: final results from a long-term open-label study. J Am Acad Child Adolesc Psychiatry. 2005;44(10):1015-23.

8 Bush G, Spencer TJ, Holmes J, Shin LM, Valera EM, Seidman LJ, et al. Functional magnetic resonance imaging of methylphenidate and placebo in attention-deficit/hyperactivity disorder during the multi-source interference task. Arch Gen Psychiatry. 2008;65(1):102-14.

9 Concerta. Tablets (Methylphenidate Hydrochloride). Common Technical Document in Japan. 2007.

10 Huang-Pollock CL, Karalunas SL, Tam H, Moore AN. Evaluating vigilance deficits in ADHD: a meta-analysis of CPT performance. J Abnorm Psychol. 2012;121(2):360-71.

11 Taghia J, Cai WD, Ryali S, Kochalka J, Nicholas J, Chen TW, et al. Uncovering hidden brain state dynamics that regulate performance and decision-making during cognition. Nat Commun. 2018;9.

12 Bishop CM. Pattern Recognition and Machine Learning. Springer: New York; 2006.

13 Everitt BS. An introduction to latent variable models. Chapman & Hall: London; 1984.

14 Ghahramani Z, Beal MJ. Variational inference for Bayesian mixtures of factor analysers. Adv Neur In. 2000;12:449-55.

15 Fox E, Sudderth E, Jordan M, Willsky A. Nonparametric Bayesian learning of switching dynamical systems. Advances in Neural Information Processing Systems. 2009;21:457-64.
